# False-positive *Legionella pneumophila* antibodies in COVID-19 patients

**DOI:** 10.1186/s40635-023-00512-8

**Published:** 2023-05-26

**Authors:** Shu-hua He, Shan Li, Li Weng

**Affiliations:** Medical Intensive Care Unit, State Key Laboratory of Complex Severe and Rare Diseases, Peking Union Medical College Hospital, Peking Union Medical College & Chinese Academy of Medical Sciences, 1 Shuai Fu Yuan, Beijing, 100730 People’s Republic of China

Dear Editor,

The number of patients with coronavirus disease 2019 (COVID-19) infection caused by severe acute respiratory syndrome coronavirus 2 (SARS-CoV-2) has increased in China since the reopening. From December 30, 2022, to January 30, 2023, a total of 23 critically ill patients with COVID-19 were consecutively admitted to a medical intensive care unit of Peking Union Medical College Hospital. We detected Immunoglobulin M (IgM) of *Legionella pneumophila* (LP) serological antibody test in 14 (14/23, 60.9%) patients. However, the subsequently confirmative investigation of both Polymerase chain reaction (PCR) (14/14, 100%) from bronchoalveolar fluid (BALF) and urine antigen test (12/12, 100%) for LP were negative.

As shown in Fig. 1A, the characteristics and managements were similar between LP-IgM positive and negative groups. Meanwhile, the PCR cycle threshold (*C*_*t*_) value (30.6 ± 4.8 vs. 26.2 ± 4.3, *p* < 0.05) and IgM titers (2.1 ± 2.8 vs. 0.3 ± 0.6, *P* < 0.05) of SARS-CoV-2 in LP-IgM positive group was higher than negative group (B).

**Fig. 1:**
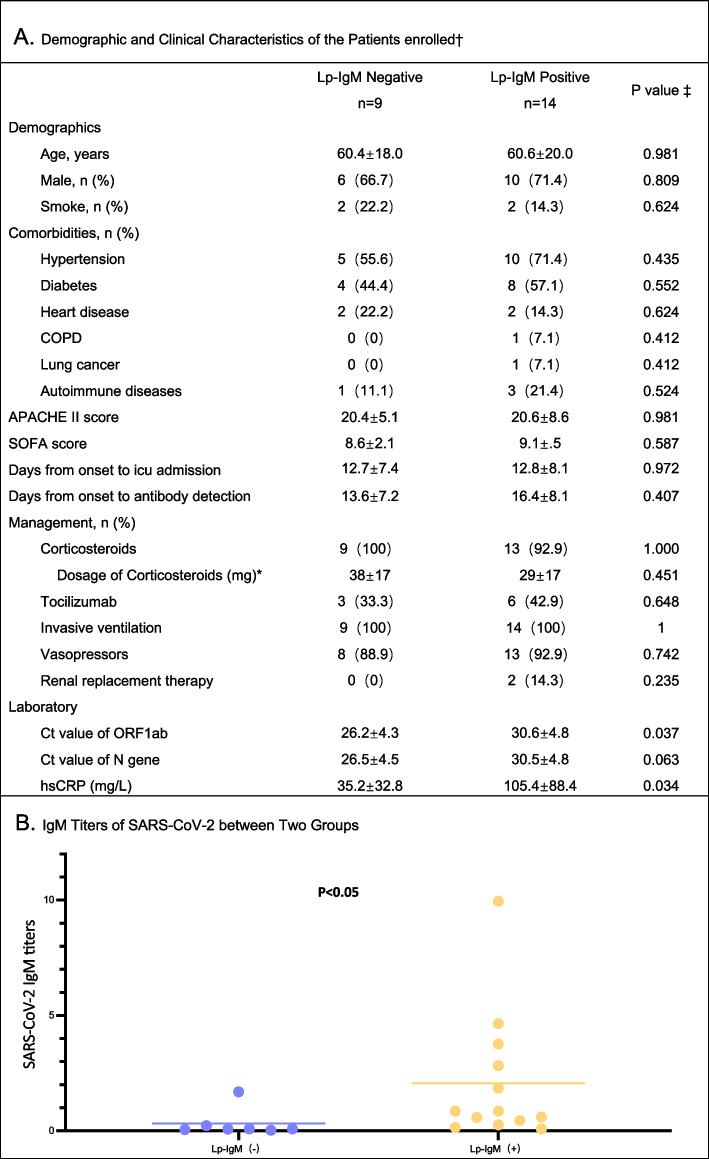
False-positive *Legionella pneumophila* antibodies in COVID-19 patients **A** shows the comparison of demographic and clinical characteristics of the enrolled patients between two groups.  **B** shows the difference of IgM titers of SARS-CoV-2 between two groups. ^†^Plus–minus values are means ± standard deviation. ^‡^The categorical variables were compared using the Chi squared test or Fisher’s exact test. The continuous variables were compared using the Mann–Whitney *U* test or *t* test. A two-sided α of less than 0.05 was considered statistically significant. *Patients who received corticosteroids on the day of LP-IgM testing (prednisone equivalent dose). *IgM* Immunoglobulin M, *LP* *Legionella pneumophila*, *APACHE* Acute Physiology and Chronic Health Evaluation, *COPD* Chronic obstructive pulmonary disease, *C*_*t*_ cycle threshold, *hsCRP* High-sensitivity C-reactive protein

Recent studies showed that the incidence of COVID-19 co-infection with LP ranged from 0.288–1.1% [1, 2] based on PCR from lower respiratory tract specimens or urine antigen testing, to 12.6–20% [3, 4]  based on immune-fluorescence or ELISA serological antibody test. To our knowledge, our study was the first report to confirm false-positive LP-IgM in COVID-19, which was similar to the cross-immune responses in previous studies [5].

Considering the potential risk of false positive results in COVID-19 patients, we suggest avoiding the immediate testing of LP-IgM or diagnosing *Legionella pneumophila* infection. Similarly, we advise against the empirical use of antibiotics such as fluoroquinolones. Instead, we recommend testing respiratory secretions DNA or urine *Legionella pneumophila* antigen for accurate diagnosis and appropriate treatment. Given the decrease in the incidence of COVID-19, the disease has become a crucial differential diagnosis, highlighting the significance of identifying patients who are admitted to the ICU with COVID-19. Our findings suggest a high positivity rate of LP-IgM in COVID-19 patients, which could serve as a potential risk factor. Therefore, clinicians should consider conducting SARS-CoV-2 testing in LP-IgM-positive patients.

## Data Availability

We have no conflicts of interests to disclose of any other funding sources.
